# Infrapatellar fat pad in the knee: is local fat good or bad for knee osteoarthritis?

**DOI:** 10.1186/ar4607

**Published:** 2014-07-09

**Authors:** Weiyu Han, Shiji Cai, Zhenhua Liu, Xingzhong Jin, Xia Wang, Benny Antony, Yuelong Cao, Dawn Aitken, Flavia Cicuttini, Graeme Jones, Changhai Ding

**Affiliations:** 1 Menzies Research Institute Tasmania, University of Tasmania, Private Bag 23, Hobart, Tasmania 7000, Australia; 2 Department of Orthopedics, the Third Affiliated Hospital of Southern Medical University, Guangzhou 510630, China; 3 Department of Epidemiology and Preventive Medicine, Monash University, Melbourne 3004, Australia

## Abstract

**Introduction:**

Recent studies regarding the infrapatellar fat pad (IPFP) mainly focus on the roles of the cells derived from the IPFP. There have been few clinical or epidemiological studies reporting on the association between the IPFP and knee osteoarthritis (OA). Our objective is to generate hypotheses regarding the associations between IPFP maximum area and knee OA measures in older adults.

**Methods:**

A total of 977 subjects between 50 and 80 years of age (mean, 62.4 years) participated in the study. Radiographic knee osteophyte and joint space narrowing (JSN) were assessed using the Osteoarthritis Research Society International atlas. T1- or T2-weighted fat suppressed magnetic resonance imaging (MRI) was utilized to assess IPFP maximum area, cartilage volume, cartilage defects, and bone marrow lesions (BMLs). Knee pain was assessed by self-administered Western Ontario McMaster Osteoarthritis Index (WOMAC) questionnaire.

**Results:**

After adjustment for potential confounders, IPFP maximum area was significantly associated with joint space narrowing (odds ratio (OR): 0.75, 95% confidence interval (CI): 0.62 to 0.91 (medial), 0.77, 95% CI: 0.62 to 0.96 (lateral)) and medial osteophytes (OR: 0.52, 95% CI: 0.35 to 0.76), knee tibial and patellar cartilage volume (β: 56.9 to 164.9 mm^3^/cm^2^, all *P* <0.001), tibial cartilage defects (OR: 0.58, 95% CI: 0.41 to 0.81 (medial), 0.53, 95% CI: 0.40-0.71 (lateral)), any BMLs (OR: 0.77, 95% CI: 0.63 to 0.94), and knee pain on a flat surface (OR: 0.79, 95% CI: 0.63 to 0.98). IPFP maximum area was negatively, but not significantly, associated with femoral cartilage defects, lateral tibiofemoral BMLs, and total knee pain or other knee pain subscales.

**Conclusion:**

IPFP maximum area is beneficially associated with radiographic OA, MRI structural pathology and knee pain on a flat surface suggesting a protective role for IPFP possibly through shock absorption. Consequently, we must pay special attention to IPFP in the clinical settings, avoiding resection of normal IPFP in knee surgery.

## Introduction

Osteoarthritis (OA), the most prevalent form of arthritis, is a common cause of chronic disability in older adults [1]. It can affect one or more joints of the body but is most common in the knees [2]. Traditionally, it is characterized by loss of articular cartilage and formation of osteophytes; however, evidence has emerged that OA involves the entire joint tissues, including the menisci, ligaments, subchondral bone, capsule, synovium, and periarticular muscle [2,3]. Although the pathogenesis of knee OA is not fully elucidated, both mechanical and metabolic factors play roles in the progression of this disease [1,2]. Age [4], female sex [5], and body mass index (BMI) [6] are well-known risk factors for knee OA.

Infrapatellar fat pad (IPFP), an intracapsular but extrasynovial structure [7], is situated in the knee under the patella, between the patellar tendon, femoral condyle and tibial plateau [8], and is structurally similar to subcutaneous adipose tissue [9]. Recent studies [10,11] mainly focus on the roles of the cells derived from IPFP, such as inflammatory cells and substance P nerve cells in OA, and consider IPFP as an active joint tissue in the initiation and progression of knee OA [8], as inflammatory cells from IPFP can produce inflammatory mediators, which are able to influence the cartilage and synovium metabolism, and substance P nerve could be an important source of pain in knee OA. IPFP is commonly resected during knee surgery; however, IPFP locates so closely to cartilage and bone surface that it may reduce the impact loading and absorb forces generated through the knee joint. So far, there have been few clinical or epidemiological studies [12] reporting the association between IPFP and knee OA measures, so the role of IPFP in knee OA is largely unknown.

Clinical features (such as joint pain) and joint structural abnormalities such as joint space narrowing (JSN), osteophytes, loss of cartilage volume, cartilage defects and bone marrow lesions (BMLs) are usually used to assess development/progression of knee OA [13]. The aim of this study was, therefore, to generate hypotheses regarding the associations between symptoms, joint structural abnormalities and IPFP area in older adults.

## Methods

### Subjects

This study was conducted as part of the Tasmanian Older Adult Cohort (TASOAC) study, an ongoing prospective, population-based study aimed at identifying the environmental, genetic, and biochemical factors associated with the development and progression of OA (assessed by both radiography and magnetic resonance imaging (MRI)). Subjects (n = 1,100) between the age of 50 and 80 years were randomly selected from the roll of electors in southern Tasmania (population, 229,000), a comprehensive population listing with an equal number of men and women. The overall response rate was 57%. Institutionalized persons and subjects with contraindications to MRI were excluded. The study was approved by the Southern Tasmanian Health and Medical Human Research Ethics Committee, and written informed consent was obtained from all participants. Self-report of diseases including asthma, cardiovascular disease, diabetes, and rheumatoid arthritis was recorded by questionnaire.

### Anthropometrics and joint pain assessment

Height was measured to the nearest 0.1 cm (with shoes, socks, and headgear removed) using a stadiometer. Weight was measured to the nearest 0.1 kg (with shoes, socks, and bulky clothing removed) by using a single pair of electronic scales (Delta Model 707, Seca, Hamburg, Germany) that were calibrated using a known weight at the beginning of each clinic. BMI (weight (kg)/height (m^2^)) was also calculated. Total body and trunk fat were measured by a Hologic dual-energy x-ray absorptiometry (DXA) scanner (Hologic Corp., Waltham, MA, USA).

The assessment of knee pain (on a flat surface, going up/down stairs, at night, sitting/lying, and standing upright) was self-administered, using the Western Ontario McMaster Osteoarthritis Index (WOMAC) with a 10-point scale from 0 (no pain, stiffness, or functional problems) to 9 (most severe) [14]. Each compartment of joint pain was summed to create a total pain score (0 to 45), and the presence of knee pain was defined as a total score or a subscale score ≥1 [15,16].

### Lower-limb muscle strength and knee radiographic assessments

We used dynamometry to measure lower-limb muscle strength twice, and took the mean as the final result, as previously described [17,18]. A standing anteroposterior semiflexed view of the right knee with 15 degrees of fixed-knee flexion was performed in all subjects, and radiographs were individually assessed for JSN and osteophytes on a scale of 0 to 3 (0 = normal and 3 = most severe) using the Osteoarthritis Research Society International (OARSI) atlas developed by Altman *et al*. [19]. We summed the osteophyte and JSN scores as the knee total radiographic OA (ROA) score; an ROA score ≥1 was used to define the presence of knee ROA, as previously described [20].

### Magnetic resonance imaging assessment

**Figure 1:**
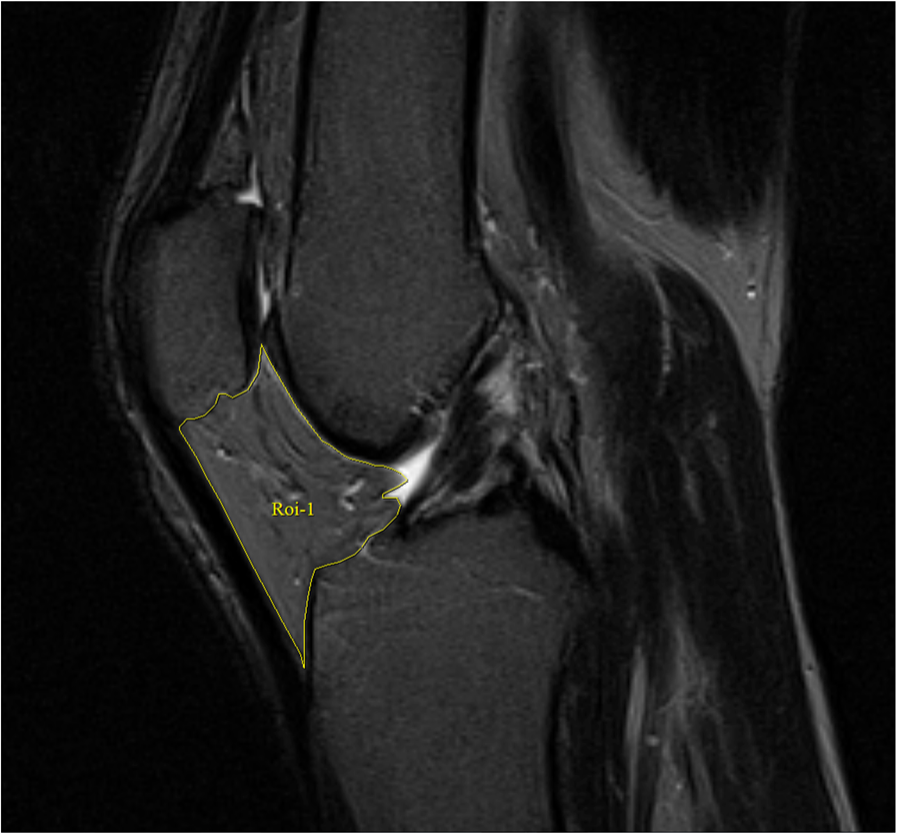
**Measurement of infrapatellar fat pad area.** The areas of infrapatellar fat pad were measured by manually drawing disarticulation contours around the infrapatellar fat pad boundaries on a section-by-section T2-weighted magnetic resonance imaging. The maximum area was selected to represent the infrapatellar fat pad area.

MRI scans of the right knees were performed at baseline. Knees were imaged in the sagittal plane on a 1.5-T whole-body magnetic resonance unit (Picker, Cleveland, OH, USA) with the use of a commercial transmit-receive extremity coil. The following image sequences were used: (1) a T1-weighted fat saturation three-dimensional gradient recall acquisition in the steady state; flip angle 30 degrees; repetition time 31 msecs; echo time 6.71 msec; field of view 16 cm; 60 partitions; 512 × 512 matrix; acquisition time 11 minutes 56 sec; one acquisition. Sagittal images were obtained at a partition thickness of 1.5 mm and an in-plane resolution of 0.31 × 0.31 (512 × 512 pixels), and (2) a T2-weighted fat saturation two-dimensional fast spin echo, flip angle 90°, repetition time 3,067 ms, echo time 112 ms, field of view 16 cm, 15 partitions, 256 × 256-pixel matrix; sagittal images were obtained at a slice thickness of 4 mm with a interslice gap of 1.0 mm.IPFP was measured by manually drawing disarticulation contours around the IPFP boundaries (Figure 1) on a section-by-section T2-weighted MR image, using the software program Osiris (University of Geneva). The maximum area was selected to represent the IPFP size. One observer graded the IPFP area on all MRI scans. The intraclass correlation coefficient (ICC) was 0.96 for intra-observer reliability (measured in 40 images by one observer), and inter-observer reliability was 0.92 (measured in 40 images by two observers).

Knee cartilage volume was determined on T1-weighted MR images with image processing on an independent work station, as previously described [17,18,21]. The total cartilage volume was divided into patellar, medial and lateral tibial cartilage volume by manually drawing disarticulation contours around the cartilage boundaries, section by section, which were then re-sampled for the final three-dimensional rendering [17,18]. The coefficients of variation (CVs) for this method in our hands were 2.1% to 2.6% [17,18].

Cartilage defects (0 to 4 scale) were assessed at the medial tibial, medial femoral, lateral tibial, lateral femoral, and patellar sites using T1-weighted images as previously described [22,23] and were further confirmed using T2-weighted images as follows: grade 0 = normal cartilage; grade 1 = focal blistering and intracartilaginous low-signal intensity area with an intact surface; grade 2 = irregularities on the surface or bottom and loss of thickness <50%; grade 3 = deep ulceration with loss of thickness >50%; grade 4 = full-thickness chondral wear with exposure of subchondral bone. The presence of cartilage defect was defined as a cartilage defect score ≥2 at one site. Intraobserver reliability was 0.89 to 0.94 and interobserver reliability was 0.85 to 0.93 [22,24]. Subchondral BMLs were defined as discrete areas of increased signal adjacent to the subcortical bone at the medial and lateral tibia and femur on T2-weighted MR images using a semiquantitative (0 to 3 scale) scoring system. The intraobserver reliability ranged between 0.89 to 1.00, as previously described [25]. Tibial plateau bone area was determined by manually measuring on axial T1-weighted MR images, as previously described [20].

Patellofemoral synovitis was graded semiquantitatively from 0 to 3 in terms of the signal alterations in IPFP on T2-weighted MR images at the superior edge of the fat pad adjacent to the patella [26]. Intraobserver reliability was assessed in 40 subjects with an ICC of 0.91.

### Serum biomarker measurements

Serum levels of leptin, IL-6 and TNF-α were measured in first 193 subjects as described previously [27,28].

### Data analysis

Partial correlation analyses were used to examine the associations between knee cartilage volume and IPFP area, and between total tibial bone area and IPFP area. Multivariable linear regression was used to analyze the associations of IFPF area (the dependent variable) with an independent variable (age, sex, height, weight, BMI, body fat, trunk fat, leg muscle strength, patellofemoral synovitis, leptin, IL-6 or TNF-α) after adjustment for the following covariates except itself: age, sex, height (not for BMI), weight (not for BMI, fat measures), tibial bone size, disease status (diabetes, cardiovascular diseases, asthma, rheumatoid arthritis) and ROA. Univariable and multivariable linear regression analyses were used to examine the associations between knee cartilage volume/total BML score (the dependent variable) and IPFP area (the independent variable) before and after adjustment for age, sex, height, weight, tibial bone size, patellofemoral synovitis and disease status, and further ROA. Univariable and multivariable binary logistic regression analyses were used to examine the associations between IPFP area (the independent variable) and a dependent variable (knee cartilage defects, knee JSN, osteophytes, tibial or femoral BMLs, or WOMAC knee pain) before or after adjustment for the same covariates. Interactions between sex or ROA and IPFP area were investigated by regressing cartilage volume (or others, for example, BMLs) on a binary (0/1) term for sex (or ROA) within IPFP, and assessed by testing the statistical significance of the coefficient of a (sex × IPFP) or a (ROA × IPFP) product term.

Adjustments for multiple testing on regression results were undertaken using the Hochberg method [29]. A *P*-value <0.05 (2-tailed) or a 95% CI not including the null point (for linear regression) or 1 (for logistic regression) was considered as statistically significant. All statistical analyses were performed using SPSS version 20.0 for Windows (SPSS Inc., Chicago, IL, USA).

## Results

A total of 977 subjects between 50 and 80 years of age (mean, 62.4 years) participated in the present study. There were no significant differences in demographic factors (age, sex, and BMI) between these participants and those excluded (n = 123) (data not shown). Characteristics of the subjects are presented in Table 1. The mean IPFP area was 7.59 cm^2^ (SD 1.18, range 4.56 to 12.14). There was a positive association (partial *r* = 0.26, *P* <0.001) between total tibial bone area and IPFP area, so all subsequent analyses were adjusted for tibial bone area.

**Table 1:** Baseline characteristics of participants (n = 977)

**Characteristic**	Values*
Age, years	62.4 (7.4)
Sex, female, n (%)	490 (50.2)
Total body fat, kg	27.8 (8.6)
Medial tibial cartilage volume, ml	2.3 (0.6)
Lateral tibial cartilage volume, ml	2.7 (0.7)
Patella cartilage volume, ml	3.2 (0.9)
Medial tibial bone area, cm^2^	20.9 (3.1)
Lateral tibial bone area, cm^2^	12.2 (2.2)
Medial joint space narrowing, n (%)	480 (53.0)
Lateral joint space narrowing, n (%)	220 (24.3)
MTF osteophytes, n (%)	65 (7.2)
LTF osteophytes, n (%)	37 (4.1)
BML present, n (%)	348 (35.6)
MTF cartilage defects, n (%)	238 (24.4)
LTF cartilage defects, n (%)	214 (22.0)
Patellar cartilage defects, n (%)	391 (40.1)
Total WOMAC knee pain, n (%)	507 (52.1)
Patellofemoral synovitis, n (%)	243 (26.8)

*Mean (SD), or number (n) of patients (%), as mentioned in the table. BMI, body mass index; WOMAC, Western Ontario and McMasters osteoarthritis index; BMLs, bone marrow lesions; MTF, medial tibiofemoral; LTF, lateral tibiofemoral.

In multivariable analyses, IPFP area was significantly and positively associated with age (β: 1.58, 95% CI: 0.80, 2.35), height (β: 6.42, 95% CI: 5.46, 7.38), weight (β: 0.90, 95% CI: 0.47, 1.34), and negatively associated with female sex (β: -42.59, 95% CI: -59.13, -26.04) after adjustment for covariates. IPFP area was neither associated with BMI, body fat, trunk fat, leg muscle strength and patellofemoral synovitis, nor with leptin, IL-6 and TNF-α (data not shown).

Table 2 describes the associations between IPFP area and radiographic OA, JSN, and osteophytes. In univariable analyses, IPFP area was significantly and negatively associated with prevalence of radiographic OA, but inconsistently associated with JSN and osteophytes. After adjustment for age, sex, height, weight, tibial bone size, patellofemoral synovitis and disease status, larger IPFP area was significantly associated with reduced radiographic OA, medial JSN, lateral JSN and medial tibiofemoral osteophytes. The association between IPFP area and lateral tibiofemoral osteophytes was consistent but not significant in the multivariable analyses. The changes in results from univariable to multivariable analyses were mainly affected by adjustment for tibial bone area.

**Table 2:** Association between IPFP area and radiographic osteoarthritis

	**Univariable**	Multivariable*
	**Odds ratio (95% CI)**	**Odds ratio (95% CI)**
Radiographic osteoarthritis	**0.88 (0.79,0.99)**	**0.75(0.62, 0.91)**
MTF joint space narrowing	**0.87 (0.77,0.97)**	**0.75 (0.62, 0.91)**
LTF joint space narrowing	0.99 (0.88,1.14)	**0.77 (0.62, 0.96)**
MTF osteophytes	0.87 (0.70,1.09)	**0.52 (0.35,0.76)**
LTF osteophytes	**1.57 (1.19,2.07)**	0.64 (0.38,1.08)

*Adjusted for age, sex, height, weight, tibial bone size, diabetes, cardiovascular diseases, asthma, rheumatoid arthritis, and patellofemoral synovitis. IPFP, infrapatellar fat pad; MTF, medial tibiofemoral; LTF, lateral tibiofemoral. Significant differences are shown in bold.

There was a positive association (partial *r* = 0.20, *P* <0.001) between total cartilage volume and IPFP area. IPFP area was significantly associated with cartilage volume in all sites in adjusted analyses; and the associations decreased in magnitude by 17 to 35% but remained significant after further adjustment for radiographic OA (all *P* <0.001, Table 3). Moreover, though IPFP area was positively associated with cartilage defects at some sites, larger IPFP area was significantly associated with reduced medial and lateral tibial cartilage defects after adjustment for covariates particularly tibial bone size, and these associations became more evident after further adjustment for radiographic OA (all *P* <0.01, Table 3). The associations of IPFP area with femoral and patellar cartilage defects were negative but did not reach significance (Table 3).

**Table 3:** Associations between IPFP area, cartilage volume and cartilage defects

	**Univariable**	Multivariable*	Multivariable**
**Cartilage volume, β (95% CI)**			
Medial tibial, ml/cm^2^	**0.27 (0.24, 0.30)**	**0.09 (0.05, 0.13)**	**0.06 (0.02, 0.10)**
Lateral tibial, ml/cm^2^	**0.34 (0.31, 0.37)**	**0.11 (0.07, 0.16)**	**0.08 (0.04, 0.13)**
Patellar, ml/cm^2^	**0.44 (0.39, 0.48)**	**0.20 (0.14, 0.26)**	**0.16 (0.10, 0.23)**
**Cartilage defects, odds ratio (95% CI)**			
Medial tibial	1.15 (0.96, 1.37)	**0.72 (0.53, 0.96)**	**0.58 (0.41, 0.81)**
Medial femoral	1.04 (0.91, 1.19)	0.98 (0.79, 1.22)	0.93 (0.74, 1.18)
Lateral tibial	1.00 (0.87, 1.15)	**0.66 (0.52, 0.84)**	**0.53 (0.40, 0.71)**
Lateral femoral	**1.22 (1.03, 1.45)**	0.84 (0.63, 1.11)	0.73 (0.53, 1.00)
Patellar	0.96 (0.86, 1.07)	0.91 (0.76, 1.09)	0.84 (0.69, 1.02)

*Adjusted for age, sex, height, weight, tibial bone size, diabetes, cardiovascular diseases, asthma, rheumatoid arthritis, and patellofemoral synovitis. **Further adjusted for radiographic osteoarthritis. Significant differences are shown in bold. ml/cm^2^, with per-cm^2^ increase in IPFP area, cartilage volume increases in ml.

IPFP area was positively associated with BMLs in univariable analyses; however, in multivariable analyses, particularly after adjustment for tibial bone area, larger IPFP area was associated with reduced BMLs in all compartments, where increasing per-cm^2^ area was significantly associated with 30% and 17% reduced odds of presence of BMLs at medial femoral and any compartments, respectively. When further adjusted for radiographic OA, the significant associations remained, and the associations with medial tibiofemoral and medial tibial BMLs became significant (Table 4). IPFP area was negatively but non-significantly associated with lateral tibiofemoral BMLs in multivariable analyses (Table 4). The association between IPFP area and total BML scores became negatively significant after adjustment for covariates and remained significant after further adjustment for radiographic OA (Table 4).

**Table 4:** Associations between IPFP area and bone marrow lesions

	**Univariable**	Multivariable*	Multivariable**
**TF bone marrow lesions, OR (95% CI)**			
Medial	1.05 (0.92,1.19)	0.83 (0.67, 1.02)	**0.74 (0.59,0.93)**
Lateral	**1.19 (1.04,1.36)**	0.93 (0.75, 1.16)	0.91 (0.72,1.16)
**Tibial bone marrow lesions, OR (95% CI)**			
Medial	1.06 (0.92,1.24)	0.87 (0.68, 1.10)	**0.74 (0.57,0.96)**
Lateral	1.17 (0.94,1.45)	0.84 (0.58,1.20)	0.79 (0.54,1.17)
**Femoral bone marrow lesions, OR (95% CI)**			
Medial	1.02 (0.86,1.22)	**0.69 (0.53, 0.92)**	**0.68 (0.51,0.91)**
Lateral	**1.19 (1.03,1.38)**	0.89 (0.69, 1.13)	0.88 (0.68,1.15)
**Any bone marrow lesions, OR (95% CI)**	**1.11 (1.00, 1.24)**	**0.83 (0.69, 0.99)**	**0.77 (0.63,0.94)**
**Bone marrow lesions total score, β (95% CI) (cm**^**2**^**/grade)**	**0.04 (0.01, 0.08)**	**-0.08 (-0.14, -0.02)**	**-0.11 (-0.17,-0.05)**

*Adjusted for age, sex, height, weight, tibial bone size, diabetes, cardiovascular diseases, asthma, rheumatoid arthritis, and patellofemoral synovitis. **Further adjusted for radiographic osteoarthritis. IPFP, infrapatellar fat pad; TF, tibiofemoral; OR, odds ratio. Significant differences are shown in bold. cm^2^/grade: with per-grade increase in bone marrow lesions, IPFP area increases in cm^2^.

IPFP area was not significantly associated with total knee pain and pain subscales in univariable analyses; however, IPFP area was significantly and negatively associated with pain when walking on a flat surface and going up/down stairs in multivariable analyses, and remained significant for pain when walking on a flat surface after further adjustment for radiographic OA (*P* <0.05, Table 5). IPFP area was not associated with other pain subscales and total knee pain score in multivariable analyses (Table 5).

**Table 5:** Associations between IPFP area and prevalent WOMAC knee pain

	**Univariable**	Multivariable*	Multivariable**
	**Odds ratio (95% CI)**	**Odds ratio (95% CI)**	**Odds ratio (95% CI)**
Total WOMAC knee pain	0.94 (0.84, 1.04)	0.84 (0.70, 1.00)	0.86 (0.71, 1.04)
Pain on flat surface	0.99 (0.88, 1.12)	**0.80 (0.65, 0.98)**	**0.79 (0.63, 0.98)**
Pain on stairs	0.93 (0.83, 1.03)	**0.81 (0.67, 0.97)**	0.83 (0.69, 1.01)
Pain in bed	0.93 (0.82, 1.04)	0.87 (0.71, 1.05)	0.83 (0.68, 1.03)
Pain when sitting	0.94 (0.83, 1.06)	0.89 (0.73, 1.09)	0.87 (0.70, 1.08)
Pain when standing	1.07 (0.95, 1.20)	1.03 (0.85, 1.26)	0.96 (0.78, 1.19)

*Adjusted for age, sex, height, weight, tibial bone size, diabetes, cardiovascular diseases, asthma, rheumatoid arthritis, and patellofemoral synovitis. **Further adjusted for radiographic osteoarthritis. WOMAC, Western Ontario and McMasters osteoarthritis index. Significant differences are shown in bold.

There were no significant interactions between sex and IPFP area or between radiographic OA and IPFP area on the outcomes (cartilage volume, cartilage defects, BMLs) (data not shown) so male and female subjects or participants with and without radiographic OA were combined for analyses. The results remained largely unchanged when subjects with rheumatoid arthritis were excluded for analyses (data not shown). All associations (except the association with knee pain) remained significant after adjustment for multiple comparisons (data not shown).

## Discussion

To our knowledge, this cross-sectional study is the first to report the significant associations between IPFP area and clinical and structural abnormalities of the knee joint in older people. We found consistent evidence that IPFP area was beneficially associated with radiographic OA, knee structural abnormalities and pain. This was independent of patellofemoral synovitis, body size, tibial bone area and other covariates, suggesting IPFP area has an important protective role in knee OA.

In a previous study, Chuckpaiwong *et al*. [12] measured IPFP volume using MRI in a cohort of 15 control subjects and 15 knee OA subjects, and reported that BMI was not significantly associated with IPFP volume in either the control or the OA group, and age was significantly and positively associated with IPFP volume in the OA group and the whole cohort. Our findings on age and BMI were consistent with this study, but we found that weight and height, measures of body size rather than obesity status, were significantly and positively associated with IPFP area. Furthermore, we found that women had a smaller IPFP area than men, and a larger tibial bone area (a measure of knee size) was associated with greater IPFP area, providing support for the construct validity of IPFP area measurement. All these factors (except for BMI) were used as adjusting variables in our analyses.

Body mass and/or fat are considered strong risk factors for knee OA. However, the role of regional fat is much less clear. The major adipose tissue in the knee joint is IPFP, and *in vitro* and animal studies have reported that IPFP can produce inflammatory cytokines and adipokines that may have detrimental effects on cartilage in knee OA [30-32]; in contrast, a meeting abstract reported that in mice, although high-fat diet increased IPFP volume, the adipocytes in the IPFP did not become hypertrophic. IPFP adipocytes may be protected from obesity-induced macrophage infiltration and inflammation, suggesting that IPFP is not a source of microphage-mediated inflammation in a diet-induced obese model of early-onset knee OA [33]. A recent study reported that medium conditioned by IPFP from end-stage OA inhibited nitric oxide (NO) production as well as matrix metalloproteinase (MMP)-1, MMP-3 and collagen type II gene expression, and thus may contribute to the inhibition of cartilage catabolism [34]. Considering the intra-articular position of the IPFP with a flexible and displaceable structure [7], IPFP is likely to absorb force and reduce overloading of the knee joint, and have a protective effect on the knee.

Chuckpaiwong *et al*. [12] reported that there was no difference in IPFP volume between control and OA groups, possibly due to a small sample size. In contrast, we found that IPFP area was significantly and beneficially associated with cartilage volume and cartilage defects in the current study. Additionally, IPFP area was associated with decreased presence of JSN, an indirect estimate of cartilage loss on radiography, in both medial and lateral tibiofemoral compartments. While these data are cross-sectional, they provide very consistent evidence in support of a protective effect of local joint fat on articular cartilage, which is opposite to the effects of systematic fat [35]. We found that IPFP area was not associated with systematic fat mass, and metabolic and inflammatory biomarkers, all of which were associated with increased knee symptoms and cartilage loss [15,27,28,36]. This suggests that systemic metabolic changes do not necessarily affect the size of IPFP.

The commonest subchondral bone abnormalities in knee OA are osteophytes and BMLs. Both are associated with knee pain [37], cartilage defects and cartilage loss [38], and need for total knee replacement [39]. In this study we reported that osteophytes and BMLs were negatively associated with IPFP area, particularly in the medial compartment. These findings further support that IPFP is protective against knee structural changes in OA.

Abnormalities of some joint structures, such as subchondral bone, capsule, ligaments, meniscus and synovium, have been associated with knee pain. It has been suggested that the sensory nerves located in these joint tissues can release substance P, calcitonin gene-related peptide (CGRP), neuropeptide Y and vasoactive intestinal peptide (VIP) into the local microenvironment, which act as the signals of pain [40]. IPFP has been regarded as an important source of pain in knee OA as it contains substance P nerve [8]; however, the association between IPFP and knee pain has not been clarified, though a previous study reported that IPFP volume was not associated with knee pain [12]. Our current study demonstrated that the IPFP area was associated with decreased presence of knee pain when walking on a flat surface and, and to a lesser extent, pain when walking upstairs/downstairs, which is opposite to what was expected, thus it had a protective effect on mechanical knee pain, which is biologically plausible.

The reasons underlying the protective effects of IPFP on joint structures and symptoms are unclear. It may be that some biochemical factors secreted from IPFP are protective, because it has been shown that medium conditioned by OA IPFP inhibits catabolic processes in cartilage [34]; it may most likely be due to the shock-absorbing nature of IPFP. As we know, biomechanical factors, especially abnormal mechanical stress/loading, play an important role in the initiation and progression of OA [41]. IPFP may have the same function as meniscus that can reduce mechanical overloading (especially in knee flexion) and absorb shock (an alteration in the environment of the joint, which can lead to cartilage degradation) through the joint. Additionally, IPFP, having the same anatomical location as the patellar ligament around the joint, may reduce instability and injury to the joint, and thus prevent the onset and progression of OA. Based on these finding, we conclude that IPFP, especially its size, plays a beneficial rather than detrimental role in the initiation or progression of OA. In the clinical setting, the IPFP is often deliberately partially or totally resected for clear visualization of the joint for the surgeons. This study suggests this may be deleterious.

The main strength of this study is that we selected participants randomly from the community, with a large sample size, and obtained both structural and symptomatic measurements. This study has several potential limitations. First, this study measured IPFP area on two-dimensional T2-weighted MRI, rather than IPFP volume on three-dimensional T1-weighted MRI. However, the boundary of IPFP on two-dimensional T2-weighted MRI is clearer than that on three-dimensional T1-weighted MRI; also IPFP area was highly correlated to IPFP volume in our analysis (*r* = 0.87) (unpublished data). IPFP volume measurement is also time-consuming. We did not measure IPFP quality, such as edema and fibrosis, which may be associated with the progression of OA. Second, measurement error may influence results. However, all measures were highly reproducible suggesting this is unlikely. Third, a large number of statistical tests have been performed, which may induce false positive results due to multiple testing; however, almost all significant associations remained after adjustment for multiple comparisons. Though the associations between IPFP area and knee pain did not pass the Hochberg correction, the consistent associations with weight-bearing knee pain subscales (walking on a flat surface and going up/down stairs) suggest the findings are biologically plausible, and may not be false positives. Last, the cross-sectional nature of this study precludes any inference about cause and effect relationships. Longitudinal studies are needed to address causality.

## Conclusions

In conclusion, IPFP maximum area is beneficially associated with radiographic OA, MRI structural pathology and knee pain on a flat surface, suggesting a protective role for IPFP, possibly through shock absorption. Consequently, we must pay special attention to IPFP in the clinical setting, avoiding resection of normal IPFP in knee surgery.

## Abbreviations

BMI: body mass index; BML: bone marrow lesion; CV: coefficient of variation; IL-6: interleukin-6; IPFP: infrapatellar fat pad; JSN: joint space narrowing; LTF: lateral tibiofemoral; MRI: magnetic resonance imaging; MTF: medial tibiofemoral; OA: osteoarthritis; OARSI: Osteoarthritis Research Society International; ROA: radiographic osteoarthritis; TF: tibiofemoral; TNF-α: tumor necrosis factor-α; WOMAC: Western Ontario McMaster Osteoarthritis Index.

## Competing interests

The authors declare that they have no competing interests.

## Authors’ contributions

CD had full access to all of the data in the study and takes responsibility for the integrity of the data and the accuracy of the data analysis. CD carried out the study design, participated in the acquisition, analysis and interpretation of data, manuscript preparation, and statistical analysis. WH participated in the acquisition, analysis and interpretation of data, manuscript preparation, and statistical analysis. SC participated in the acquisition of data, manuscript preparation, and statistical analysis. ZL participated in the analysis and interpretation of data, manuscript preparation, and statistical analysis. XJ participated in the acquisition, analysis and interpretation of data, and manuscript preparation. XW participated in the acquisition of data, manuscript preparation, and statistical analysis. BA participated in the acquisition, analysis and interpretation of data, and manuscript preparation. YC participated in the acquisition of data, manuscript preparation, and statistical analysis. DA participated in the acquisition of data, and manuscript preparation. FC participated in the study design, and manuscript preparation. GJ participated in the study design, acquisition, analysis and interpretation of data, and manuscript preparation. All authors read and approved the final manuscript.
